# Drug Resistance Mechanisms of Acute Myeloid Leukemia Stem Cells

**DOI:** 10.3389/fonc.2022.896426

**Published:** 2022-07-05

**Authors:** Jialan Niu, Danyue Peng, Lingbo Liu

**Affiliations:** Institute of Hematology, Union Hospital, Tongji Medical College, Huazhong University of Science and Technology, Wuhan, China

**Keywords:** acute myeloid leukemia, leukemia stem cells, drug resistance, resistant mechanisms, LSCs remolding, clonal heterogeneity

## Abstract

Acute myeloid leukemia (AML) is a polyclonal and heterogeneous hematological malignancy. Relapse and refractory after induction chemotherapy are still challenges for curing AML. Leukemia stem cells (LSCs), accepted to originate from hematopoietic stem/precursor cells, are the main root of leukemogenesis and drug resistance. LSCs are dynamic derivations and possess various elusive resistance mechanisms. In this review, we summarized different primary resistance and remolding mechanisms of LSCs after chemotherapy, as well as the indispensable role of the bone marrow microenvironment on LSCs resistance. Through a detailed and comprehensive review of the spectacle of LSCs resistance, it can provide better strategies for future researches on eradicating LSCs and clinical treatment of AML.

## Introduction

Acute myeloid leukemia (AML) is a hematopoietic disease characterized by malignant proliferation of myeloid stem/progenitor cells and differentiation arrest. Standard intensive chemotherapy-based regimens remain central in inducing complete remission (CR) in AML, with CR rates of 60–85% for adults younger than 60 years of age and 40–60% for older patients aged ≥60 years (1). Furthermore, with an advanced understanding of the pathogenesis and drug-resistant mechanisms, new therapeutic agents in clinical development target abnormal karyotypes, such as FMS-like tyrosine kinase 3 (FLT3) inhibitors (2), isocitrate dehydrogenase 1 or 2 (IDH1/2) inhibitors (3, 4), and target effector molecules of apoptosis, metabolism, epigenetics, stemness, and immune escape, such as B-cell lymphoma 2 (BCL2) inhibitors (5, 6), hypomethylating agents (HMA) (7), smoothened (SMO) inhibitors (8), CD33 monoclonal antibody (9), have improved the overall survival of patients, especially elderly patients with AML who are not candidates for intensive chemotherapy (10). However, there are still induction failures in some patients with AML with high-risk karyotypes, and short- or long-term relapse occurs after complete remission in most patients, based on different relapse/refractory mechanisms (11).

**Figure 1:**
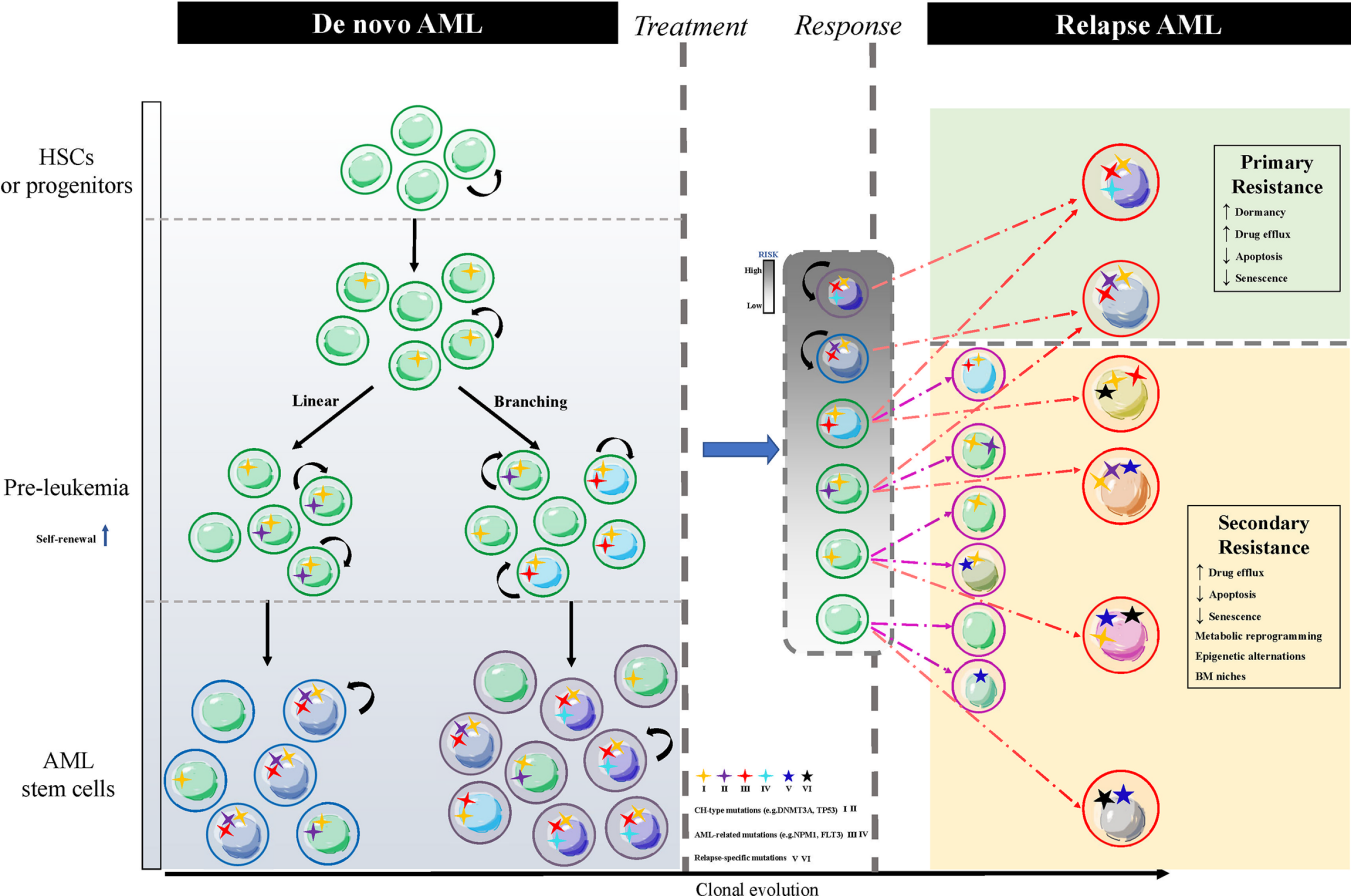
The schematic of LSCs clonal diversification resulting in AML relapse. The normal HSCs or progenitor cells carry CH-type mutations without proliferative potentials, such as ‘DTA’ mutations, denoted by the number I, and convert to pre-leukemia HSCs or progenitor cells with enhanced self-renewal through acquiring additional genetic mutations, which are divided into linear and branching patterns of clonal evolution. The former stepwise acquires CH-type mutations like TP53, IDH1/2 and AML-related mutations like FLT3, NPM1. The latter parallelly acquires CH-type mutations and AML-related mutations in different cell populations. Different colors of nuclei show heterogeneous LSCs clones. Residual leukemia cells (grey box) after treatment include dominant clones LSCs and subclones leukemia cells. Relapsed AML arises from these two types of cells corresponding to the primary resistance (green box) of LSCs and the secondary resistance (yellow box) of reprogramming leukemia cells *via* acquiring a new relapse-driven clone. AML, acute myeloid leukemia; LSCs, AML stem cells; HSCs, hematopoietic stem cells; CH, clonal hematopoiesis; BM, bone marrow; ‘DTA’ mutations, mutations in DNMT3A, TET2 and ASXL1.

Lapidot and Bonnet et al. successively identified a rare population of leukemia cells capable of initiating leukemia when transplanted into severe combined immune-deficient (SCID) mice. The rare subset that marked the CD34^+^CD38^-^ population was the same as that of normal hematopoietic stem cells (HSCs), and was defined as leukemia stem cells (LSCs). These LSCs possessed self-renewal and unlimited proliferation potential (12, 13). Several subsequent studies also confirmed the existence of this cell population (14–17). The established views described that current chemotherapy drugs could only eradicate most of the AML blast cells, but not LSCs, and LSCs were the root of AML relapse (18, 19). Furthermore, there were also treatment-resistant cells carrying leukemia clones with different characteristics from the originally diagnosed LSCs (19–21). Chemotherapy-induced leukemia repopulating cells, some of which derive from LSCs present intrinsic mechanisms of chemotherapy resistance, and are termed primary resistances while others derive from leukemia cells that regain stemness under chemotherapy stress, and are termed secondary resistance mechanisms (22). Therefore, identifying both primary and secondary mechanisms of chemotherapy resistance is essential for the targeted elimination of LSCs and ultimately the cure of AML. This review mainly summarizes the primary and secondary resistance mechanisms of different leukemia clones identified in recent years (**Figure 1**), including (1) the inherent dormancy of LSCs that protects them from cell cycle-specific agents (CCSA) ;(2) the overexpression of multiple ATP-binding cassettes (ABC) transporters in LSCs transports cytotoxic drugs out of the cell ;(3) LSCs with the defects in apoptotic signals lead to chemotherapy resistance ;(4) senescent resistance mechanisms also result in chemotherapeutic failure ;(5) metabolic reprogramming of LSCs allows them to adjust to energy changes ;(6) epigenetic alternations and reprogramming refresh the stemness of LSCs; and (7) bone marrow (BM) niches that provide a sanctuary for LSCs from therapeutic drugs.

## Dormancy of LSCs and Drug Resistance

In short, the dormant cancer cells are defined as being in a state of reversible nonproliferation, which includes both the quiescent cell populations in the primary tumor and residual tumor cells after chemotherapy-induced stress, both are closely associated with tumor recurrence. Thus, dormant cancer cells include not only cancer stem cells (CSCs) but also cancer cells that have acquired stemness (23). In AML patients, CD34^+^CD38^-^ LSCs are enriched with stemness-related genes and persist after chemotherapy, and are closely correlated with a poor prognosis (14, 18, 24, 25). LSCs, derived from hematopoietic stem cells or progenitor cells, acquire self-renewal capacity and stemness in the process of clone formation and often remain in a quiescent state (26). LSCs are in the G0 phase prior to transplantation into nonobese diabetic(NOD)-SCID mice, and some remain quiescent and self-renewal after several consecutive transplants (27). With different methods such as the use of granulocyte colony-stimulating factor (G-CSF) to induce dormant LSCs into the cell cycle, initially slow-proliferating LSCs become susceptible to chemotherapy (28). Generally, AML recurrences are mainly attributed to quiescent LSCs, because conventional CCSA only act on proliferating cells but not on quiescent LSCs. Herein, we mainly describe the regulation of LSCs quiescence by cellular molecular mechanisms and their surrounding microenvironment.

**Figure 2:**
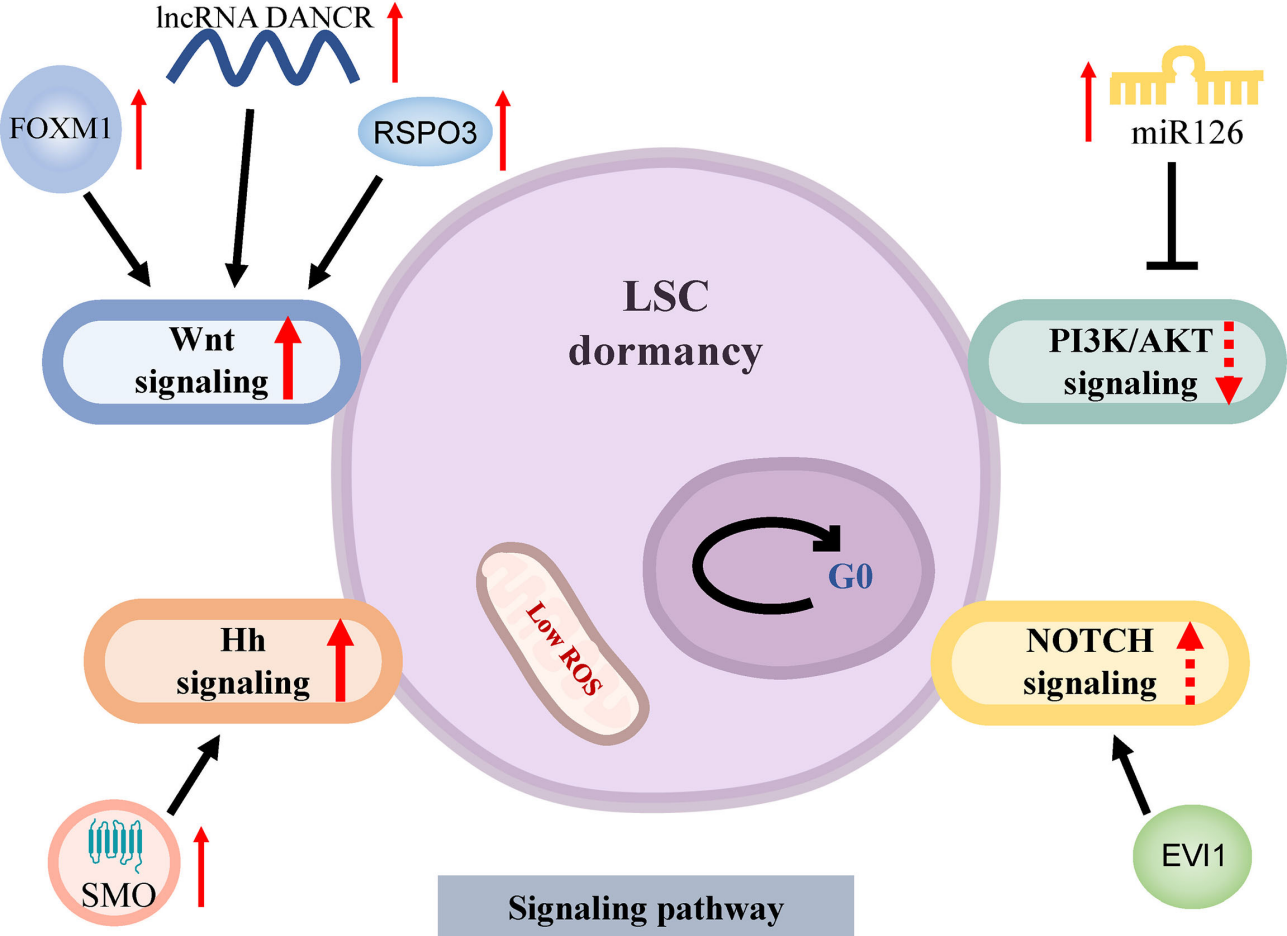
Signaling pathways of dormancy in LSCs. LSCs arrest the cell cycle at G0 phase in a low level of ROS. The Wnt signaling pathway is activated through overexpression of FOXM1 which stabilizes β-catenin, up-regulated RSPO3 by activating β-catenin and increased lncRNA DANCR which upregulates MYC expression to induce LSCs dormancy. The repression of PI3K/AKT signaling pathway by miR126 mediates the stemness of LSCs and drug resistance. The abnormal activation of the Hh and NOTCH signaling pathways plays an important role in maintaining LSCs quiescence. AML, acute myeloid leukemia; LSCs, AML stem cells; ROS, reactive oxygen species; Hh, Hedgehog; SMO, smoothened; EVI1, Ecotropic virus integration site 1.

The activation of the Wnt signaling pathway is one of the most important pathways for the maintenance of LSCs quiescence (**Figure 2**). Wnt/β-catenin activation is required for the conversion of HSCs as well as relatively mature granulocyte-macrophage progenitor cells (GMPs) into LSCs, regulates LSCs stemness, self-renewal and proliferation (29). R-spondin 3 (RSPO3) activates β-catenin by binding to LGR4 (leucine-rich repeat-containing G protein-coupled receptors) high-expressed on the surface of LSCs, further promotes the expression of stemness and self-renewal genes such as MYC, CCND1 and HOXA clusters. The anti-RSPO3 antibody can reliably debilitate the ability of LSCs to restart leukemia through serial transplantation in mice (30). Recently, it has been discovered that the transcription factor FOXM1 activates the Wnt/β-catenin molecular pathway through stabilizing β-catenin expression to mediate quiescence and self-renewal of MLL-AF9-LSCs (31). In particular, FOXM1 inhibitors do not significantly alter downstream Nurr1 expression [one of the most important molecules regulating HSCs quiescence (32)] and have a lower impact on HSCs, providing a therapeutic window for the targeted elimination of LSCs. Other studies have shown that the long non-coding RNA (lncRNA)-DANCR (differentiation antagonizing non-protein coding RNA) is involved in the regulation of the Wnt signaling pathway. LncRNA DANCR is relatively highly expressed in CD34^+^ AML primary cells, and knocking down of lncRNA DANCR expression reduces the quiescence and self-renewal of LSCs *in vitro* and *in vivo* by down-regulating MYC expression, but not that of normal hematopoietic stem and progenitor cells (HSPCs) (33). The differential regulation of MYC expression by lncRNA DANCR illustrates the important role of the Wnt signaling pathway in LSCs resistance. Although lncRNA DANCR is differentially regulated in HSCs and LSCs, the resistance mechanisms in LSCs require a thorough understanding. Further research is needed on how to clinically inhibit the Wnt signaling pathway to provide a therapeutic window for the targeted clearance of LSCs (34).

The other important mechanism of quiescent maintenance of LSCs involves the PI3K/AKT signaling pathway, LSCs rely on the relative low-activity AKT to keep quiescent (**Figure 2**). PI3K/AKT signaling pathway is usually activated during leukemogenesis to promote cell proliferation and is associated with specific genetic alterations like mutations of FLT3 and NPM1 (35). Thus, the state of LSCs depends on rigorous AKT regulation. Micro(miR)-126 is an important negative regulator of the PI3K/AKT pathway in LSCs. The overexpression of miR-126 in LSCs reduces AKT activity by targeted down-regulation of CDK3, a regulator of G0/G1 conversion, to maintain cell cycle quiescence, improving stemness and resistance to chemotherapy (36). Recent studies have shown that miR-126 is highly expressed in the endothelial cells (ECs) surrounding BM arterioles relative to sinusoids. In the FLT3-ITD^+^ AML mouse model, treatment with tyrosine kinase inhibitors **(**TKIs) inhibited the AML-mediated remodeling of arterioles to sinusoids, resulting in the increase of miR-126 in the BM, which further enhanced LSCs quiescence, whereas, the inhibition of miR-126 can effectively eradicate LSCs and prolong survival (37). This may partly explain the greater therapeutic resistance of AML after relapse. Altogether, these findings suggest that miR-126 has a great potential in targeting quiescent LSCs.

Other signal pathways involved in LSCs quiescence are the Hedgehog (Hh) and NOTCH pathways (**Figure 2**). The Hh pathway plays an important role in maintaining quiescence, self-renewal, survival, and chemoresistance of BCR-ABL^+^ LSCs (38). The abnormal activation of the Hh signaling pathway has been observed in AML (39). The transmembrane protein smoothened (SMO), an essential regulator of Hh signal transduction, is highly expressed in CD34^+^ leukemia cells and can activate downstream glioma-associated oncoprotein (GLI) transcription factors to maintain the dormancy and chemoresistance of LSCs (40). Preclinical studies found that PF-913, an inhibitor of SMO, can decrease the proportion of CD34^+^ leukemia cells, induce dormant LSCs into the cell cycle, reduce leukemia initiation, and exert synergistic effects with cytarabine (AraC) (41). Phase II trial of glasdegib (SMO inhibitor) plus conventional chemotherapy in patients with AML achieved a better complete remission (CR) rate and prolonged overall survival (8) (**Table 1**). On the other hand, NOTCH signaling pathway can also drive the expression of stemness-related genes and maintain the pool of LSCs. LSCs with high expression of Ecotropic virus integration site 1 (EVI1) were enriched in G0 phase *in vivo* and *in vitro*, and all-trans retinoic acid (atRA) further enhanced LSCs quiescence in an EVI1-dependent manner, and EVI1 and atRA synchronously activate NOTCH signal pathway through targeting downstream molecule NOTCH4 (57). Paradoxically, the finding by Lobry showed that activation of the NOTCH signaling pathway induced cell cycle arrest and apoptosis in LSCs with MLL-AF9 fusion, in part due to Tet methylcytosine dioxygenase 2 (TET2)-mediated epigenetic alterations (58). As a result, precise modulation of NOTCH threshold to induce LSCs entry into the cell cycle and jointly promote AML blast clearance may be an interesting direction.

**Table 1:** Clinical trials of targeted therapies in AML.

Targeting mechanism	Agents	Patient Population	Eliminating LSCs	Combination therapy	ClinicalTrials.gov Identifiers
*Self-renewal Signaling*					
Wnt	CWP232291 (Degradation of β-catenin)	R/R AML	Yes (29)	Not	NCT01398462
	PRI-724 (CBP inhibitor)	AML		Not/Dasatinib/LDAC	NCT01606579
PI3K/AKT	BEZ235 (Dual PI3K/mTOR inhibitor)	Refractory AML	Yes (35)	Not	NCT01756118
	GSK2141795 (AKT inhibitor)	RAS-mutated R/R AML		Trametinib	NCT01907815^#^
	Everolimus (mTOR inhibitor)	AML		Midostaurin	NCT00544999
		Relapsed AML		Cytarabine and daunorubicin	NCT 01074086*
	Rapamycin (mTOR inhibitor)	AML		Decitabine	NCT02109744*
Hh	Glasdegib (SMO inhibitor)	de novo/relapsed AML;	Yes (38)	LDAC/Decitabine	NCT01546038*
		de novo AML		Azacitidine	NCT02367456*
		AML with MDS-related changes/therapy-related AML		CPX-351	NCT04231851
*ABC transporters*					
P-gp	Valspodar	de novo AML	Potential (42)	Cytarabine, daunorubicin and etoposide	NCT00006363^#^
	Zosuquidar	de novo AML		Cytarabine and daunorubicin	NCT00046930^#^
*Regulators of apoptosis and senescence resistance*					
BCL2	Venetoclax	de novo AML/ R/R AML	Yes (43)	Milademetan tosylate and LDAC	NCT03634228
		AML		CC-486	NCT05287568
		AML		ASTX727	NCT04657081
		R/R AML		Azacitidine and lintuzumab-Ac225	NCT03867682
		R/R AML		Alvocidib	NCT03441555
		R/R AML		Dinaciclib	NCT03484520
		de novo AML/ R/R AML		FLAG-IDA	NCT03214562*
		de novo AML		CLIA regimen	NCT02115295*
		AML		Sabatolimab and azacitidine	NCT04150029
		R/R AML		Gilteritinib	NCT03625505
MCL1	MIK665	R/R AML	Yes (44)	VOB560	NCT04702425
		AML		Azacitidine	NCT04629443
		AML		Venetoclax	NCT03672695
	PRT1419	R/R AML	Yes (45)	Not	NCT05107856
	AMG 176	R/R AML	Yes (46)	Itraconazole/ Azacitidine	NCT02675452
CDKs	Alvocidib	de novo AML	Yes (47)	Cytarabine and daunorubicin	NCT03298984*
P53-MDM2	Idasanutlin	R/R AML	Yes (48)	Cytarabine	NCT02545283
	KRT-232	de novo AML/ R/R AML		LDAC/Decitabine	NCT04113616
	APG-115	de novo AML/ R/R AML		Azacitidine/Cytarabine	NCT03634228
	HDM201	AML		Venetoclax and azacitidine	NCT05155709
P53	Arsenic Trioxide	p53-mutated AML	Yes (49)	Decitabine	NCT03855371
	Eprenetapopt	p53-mutated AML	Yes (50)	Azacitidine	NCT03588078*
*Metabolism*					
IDH1	Ivosidenib	de novo, mutant-IDH1 AML	Yes (51)	Azacitidine	NCT02677922*
		R/R, mutant-IDH1 AML		Azacitidine	NCT04250051
IDH2	Enasidenib	de novo, mutant-IDH2 AML		Azacitidine	NCT02677922*
		R/R, mutant-IDH2 AML		Azacitidine	NCT03683433
IDH1/IDH2	Ivosidenib/Enasidenib	de novo, mutant- IDH1/IDH2 AML		Intensive chemotherapy	NCT02632708
*Epigenetics*					
KDM1A	Iadademstat	AML	Yes (52)	Azacitidine	EudraCT 2018-000482-36*
HDAC	Panobinostat	de novo AML	Yes (53)	Intensive chemotherapy	(54)*
		de novo AML		Idarubicin and cytarabine	NCT01242774*
	Valproic acid	AML		ATRA and Decitabine	NCT00867672^#^
		AML		ATRA and cytarabine	NCT00995332*
	AR-42	AML		Decitabine	NCT01798901
*BM niche*					
CXCL12/CXCR4	CX-01	R/R AML	Yes (55)	Azacitidine	NCT02995655*
	Plerixafor	R/R AML		mitoxantrone, etoposide and cytarabine	NCT00512252*
TIM3	Sabatolimab	de novo AML/ R/R AML	Yes (56)	Magrolimab and azacitidine/not	NCT05367401
		AML		Venetoclax and azacitidine	NCT04150029

AML, acute myeloid leukemia; LSCs, leukemia stem cells; CPX-351, liposomal cytarabine and daunorubicin at a fixed 5:1 molar ratio; R/R, relapsed or refractory; CBP, CREB-binding protein; LDAC, low-dose cytarabine; MDS, myelodysplastic syndrome; CC-486, oral azacytidine; Lintuzumab-Ac225, humanized CD33 antibody; FLAG-IDA, fludarabine, cytarabine, granulocyte colony-stimulating factor (G-CSF), and idarubicin; ASTX727, decitabine and cedazuridine; CLIA regimen, cladribine, high-dose cytarabine and idarubicin; VOB560, BCL-2 inhibitor; ATRA, all-trans retinoic acid. * active clinical activity; ^#^no clinical activity.

In addition, the BM microenvironment also plays a vital role in maintaining LSCs quiescence. The interaction of the CXCR4/CXCL12 axis induced the localization of LSCs in the endosteal region where LSCs could maintain a quiescent state, which was closely related to resistance to chemotherapy and adverse prognosis (55). Hypoxia is essential for HSCs dormancy based on the regulation of hypoxia-inducible factors (HIFs) (59). However, the role of HIFs in LSCs is contradictory (60). The expression of HIF-1α in LSCs was increased. HIF-1α deletion led to the elimination of LSCs (61), in contrast, HIF-1α deletion promoted the conversion of pre-leukemic cells to LSCs, and HIF-1α knockout in MLL-AF9 mice showed LSCs increase and AML progression after chemotherapy (62). Recently, reactive oxygen species (ROS) have been proposed to represent the LSCs population (43). Cell populations with low ROS were rich in quiescent LSCs, which was associated with drug resistance/recurrence, compared to cell populations with high ROS (63). Eimear OR et al. simulated BM-induced dormancy of KG1a cells *in vitro* by using a hydrogel-based layered co-culture system based on bone mesenchymal stem cells (BMSCs), adding TNFβ-1 and HIFs (64). This model is valuable for targeted drugs screening to treat dormant LSCs in the microenvironment. The regulation mechanisms of BM on the quiescence of LSCs need to be further studied. The humanized mesenchymal niche model system established by Waclawiczek et al. is prospective to study the crosstalk between MSCs, LSCs and normal hematopoiesis (65).

The maintenance of LSCs quiescence and self-renewal depend on a complex intracellular molecular signaling network, which overall maintains low expression of cell cycle-related genes and proliferation-related genes, with elusive crosstalk between various stemness-related pathways. It was found that GLI1 can promote AML cell proliferation directly through PI3K signaling pathway and can be reversed by AKT inhibitors (66). AKT can enhance FOXM1 expression and form a positive feedback to enhance venetoclax resistance in FLT3 wild-type AML (67). Therefore, FOXM1 inhibitors may be a promising therapeutic strategy due to their dual signaling pathway regulations. Similarly, it was found that the combination of inhibitors targeting PI3K/AKT/mTOR signaling pathway and atRA can almost eliminate cellular MYC to drastically kill leukemia cells of different AML subtypes (68). Additionally, the crosstalk between the Wnt and NOTCH pathways has also been reported. Kang YA et al. verified that NOTCH and Wnt signal cooperatively maintained HSCs lineage differentiation. And it also demonstrated that LSCs characteristically maintain high levels of Wnt and low levels of NOTCH, and that restoring those dysregulated pathway activities can inhibit myeloid differentiation and delay leukemia progression (69). These results present the possibility of strong crosstalk in cell-cycle regulation between both pathways. Furthermore, due to the high heterogeneity within and between patients, there is no single indicator to identify LSCs. Data have shown multiple candidate surface markers including CD123 (70), CD33 (71), CD96 (72), TIM-3 (73), CD44 (74), CLL-1 (75), c-MPL (76), MHDA2 (77), CD25 and CD32 (24) that play a significant role in the stemness properties of LSCs. Of these, CD25, CD32, CD123, c-MPL, and MHDA2 are expressed in quiescent human LSCs, and targeting these surface markers may facilitate the removal of dormant LSCs. A recent study tracking the leukemia cells of AML patients before and after chemotherapy found that CD34^+^CD38^-^ LSCs and AML blasts were equally eliminated, and LSCs no longer remained quiescent after chemotherapy (21, 63). Although chemotherapy may promote the evolution of some clones, quiescence or dormancy is not sufficient to protect LSCs from chemotherapy-induced cell death (78).

## ABC Transporter Family and AML Drug Resistance

**Figure 3:**
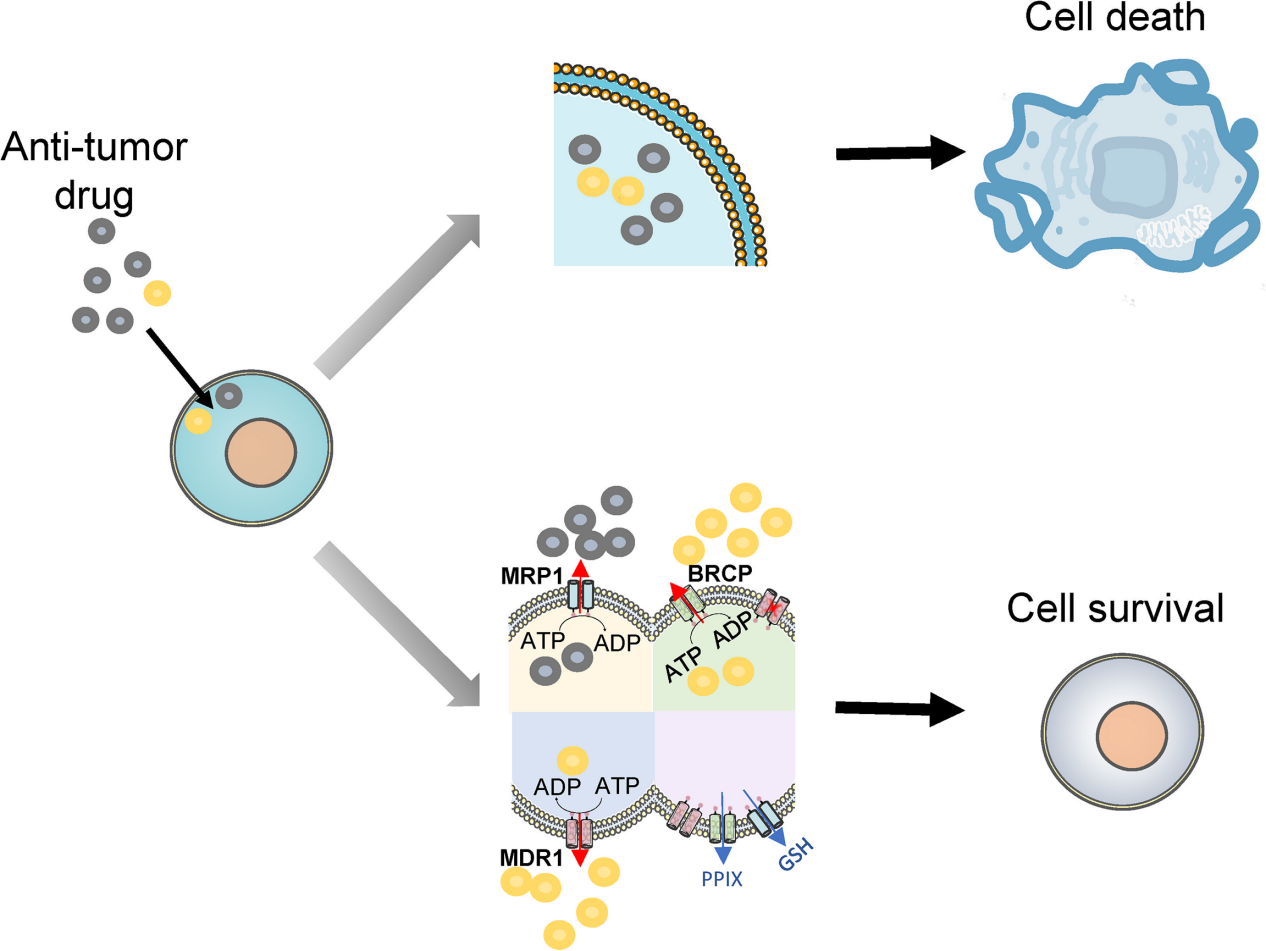
Drug efflux and drug resistance. Multiple anti-tumor drugs enter to kill leukemia cells (upper part), but LSCs upregulate ABC transporters to efflux drugs to overcome the cytotoxic effects of multiple chemotherapeutic drugs, even single blocking of MDR1 does not significantly improve chemotherapy outcomes. Additionally, MRP1 mediates the ATP-dependent efflux of GSH to reduce cellular oxidative stress. BRCP transports excessive PPIX out of the cell to maintain porphyrin homeostasis (lower part). MRP1, multidrug resistance-associated protein 1; MDR1, multidrug resistance gene 1; BRCP, breast cancer resistance protein; PPIX, protoporphyrin IX; GSH, glutathione; ATP, adenosine triphosphate; ADP, adenosine diphosphate.

A vital mechanism of multiple drug resistance in tumor cells is the transport of cytotoxic drugs outside cells to reduce DNA damage. The ABC transporter family, which currently consists of 48 members, pumps out exogenous substances *via* ATP-dependent membrane transport pathways (79). A variety of ABC transporters are highly expressed in AML cells, and are closely associated with adverse chemotherapy responses in AML patients. Consecutively, follow-up studies revealed that LSCs also have higher expression of different ABC transporters (42). Therefore, targeting ABC transporters has a positive impact on enhancing the chemosensitivity of AML. ABC transporters showing the greatest correlation with AML chemotherapy resistance are permeability glycoprotein (P-gp), breast cancer resistance protein (BCRP), and multidrug resistance-associated protein 1 (MRP1), respectively, where P-gp and BCRP are the most frequently expressed (**Figure 3**).

The best-characterized ABC transporter is P-gp, which is encoded by the multidrug resistance gene 1 (MDR1 or ABCB1). P-gp confers resistance to chemotherapeutic drugs such as doxorubicin, daunorubicin, vincristine, mitoxantrone, and methotrexate (80). Numerous studies have confirmed that leukemia cells overexpressed P-gp, which was strongly associated with the poor prognosis in AML (81–83). Subsequent studies have found that the association was age-dependent, which was more pronounced in patients older than 60 years than in young adults with AML (84). Furthermore, de Figueredo et al. reported that compared with the relatively mature CD34^+^CD38^−^CD123^−^ cells, the CD34^+^CD38^−^CD123^+^ subset had a higher P-gp, while CD34^+^CD38^−^CD123^−^ cells had an increased P-gp expression than the CD34^+^CD38^+^ subset (85). However, the study found that the use of P-gp inhibitors, such as zosuquidar or cyclosporine, failed to improve the CR rate and OS of AML patients (86). The possible cause may be that AML stem cells express multiple ABC transporters, hence, single blocking of a certain ABC transporter does not significantly improve chemotherapy outcomes. In addition, adriamycin stress can increase the expression of ABCB1 enhancers, and this adaptive change of ABCB1 mediated drug resistance in AML (87).

Another important ABC transporter, BCRP, encoded by the ABCG2 on chromosome 4, has been linked to drug resistance in the treatment of AML with chemotherapeutic agents such as doxorubicin, daunorubicin, vincristine, mitoxantrone, and methotrexate (80). Many studies have indicated that higher levels of BCRP expression were associated with lower survival rates of AML, and increased expression of BCRP in patients is observed in patients that fail to achieve CR after chemotherapy (82, 88, 89). Researchers also found that the expression of BCRP has correlated with the expression of side population (SP) cells, a stem cell-enriched fraction, with obvious potential for both self-renewal and proliferation (90). Raaijmakers et al. found that expression of BCRP was predominant in LSCs and blocking of BCRP-mediated drug export by the fumitremorgin C analog KO143 resulted in increased intracellular mitoxantrone accumulation in these cells (91). Therefore, targeting ABCG2 may be a promising therapy to eliminate LSCs.

The ABC-C subfamily encodes multidrug resistance-associated proteins (MRP), of which MRP1 was the first MRP molecule to be discovered. MRP1 is overexpressed in many multidrug-resistant cancer cell lines and confers an extensive drug resistance phenotype, which can develop resistance to multiple chemotherapy agents in AML (80). Previous studies have reported a correlation between MRP1 expression and the prognosis of AML, but the results remain controversial. Some studies have shown that high expression of MRP1 is associated with poor prognosis in AML (83, 92), contrarily, some researchers have found no such correlation (93, 94). There are several possible explanations such as the different methods used to study MRP1 expression, different treatment regimens, and patient characteristics which impact on MRP1 expression. The inconsistency of these findings has also suggested that MRP1is not a major determinant in drug resistance in AML.

Overall, LSCs exhibit a much higher expression of ABC transporters than mature cell populations, which results in chemotherapy resistance (95). Noteworthily, ABC transporters also act as efflux transporters of biomolecules relevant to tumor resistance. MRP1 mediates the ATP-dependent efflux of glutathione (GSH) to reduce cellular oxidative stress (96). BRCP transports excessive protoporphyrin IX (PPIX) out of the cell to maintain porphyrin homeostasis (97). However, clinical trials evaluating the efficacy of ABC transporters-inhibitors have been unsatisfactory. The main reason may be that HSCs also have a high expression of various ABC transporters, as demonstrated by Zhou et al. Mice models engineered to knock-out ABCB1 or ABCG2 genes are particularly sensitive to drugs such as mitoxantrone and vinblastine, so the expression of these genes may represent a protective mechanism for HSCs (98). Inhibitors of ABC transporters may reduce the chemoresistance of leukemia primitive populations and LSCs, but may also render HSCs more sensitive to chemotherapeutic drugs, which compromises normal hematopoietic function. Co-expression or activity of ABC transporters is also particularly important in chemotherapy resistance in AML patients. ABCB1 and ABCG2 mRNA co-expression was significantly associated with higher age, increased CD34 expression and poor prognosis (99). Robinson et al. developed that overexpressing both ABCB1 and ABCG2 *in vitro* not only exerted the function of substrate transport independently but also additively transport new substrate molecules (100). Therefore, it is of great significance to find ABC transporters that are specifically expressed by LSCs.

## Apoptosis Resistance of LSCs is Associated With Drug Resistance

The induction of apoptosis of AML cells by chemotherapy is an effective approach to kill malignant cells. However, recent studies have shown that multiple anti-apoptotic mechanisms exist in LSCs. Defects of apoptosis-related signaling pathways represent an integral mechanism for treatment resistance in AML. The principal apoptosis-regulatory molecules are B-cell lymphoma 2 (BCL-2), myeloid cell leukemia sequence 1 (MCL1) and p53, mutations or abnormalities of which can induce apoptotic resistance of LSCs.

BCL-2, the foremost anti-apoptotic protein in hematological malignancies, is significantly up-regulated in lymphoma, multiple myeloma, and AML (101). BCL-2 can stabilize mitochondria and prevent the activation of pro-apoptotic proteins. The expression of BCL-2 in different AML subtypes is irregular, with much higher levels in M1 and M2 subtypes than in M3, M4, and M5 subtypes (102). Furthermore, higher expression of BCL-2 is associated with a worse response to chemotherapy and inferior survival of patients, indicating that BCL-2 is an important factor contributing to the prognosis of AML (102, 103). BCL-2 is markedly up-regulated in LSCs, and its inhibition can restrain oxidative phosphorylation and selectively eradicate quiescent LSCs (43). Venetoclax, which specifically targets BCL-2, achieves a wide range of anti-leukemia capabilities, partly due to the decreasing apoptotic threshold of AML cells (104). MCL1 is another antiapoptotic protein involved in leukemia cell survival. Overexpression of MCL1 has been associated with drug resistance and a poor prognosis of AML (105). The increased expression of MCL1 is also another important cause of venetoclax-based resistance (106, 107). Several MCL1 inhibitors have currently been evaluated in clinical trials, and are one of the promising molecules for targeted AML treatment and detailed examples will be discussed below. Similarly, the inhibitor of cyclin-dependent kinase 9 (CDK9) induced AML apoptosis by down-regulating the expression of MCL1 and demonstrated anti-leukemia and clinical activity in refractory and relapsed (R/R) AML (47, 108) (**Table 1**).

**Figure 4:**
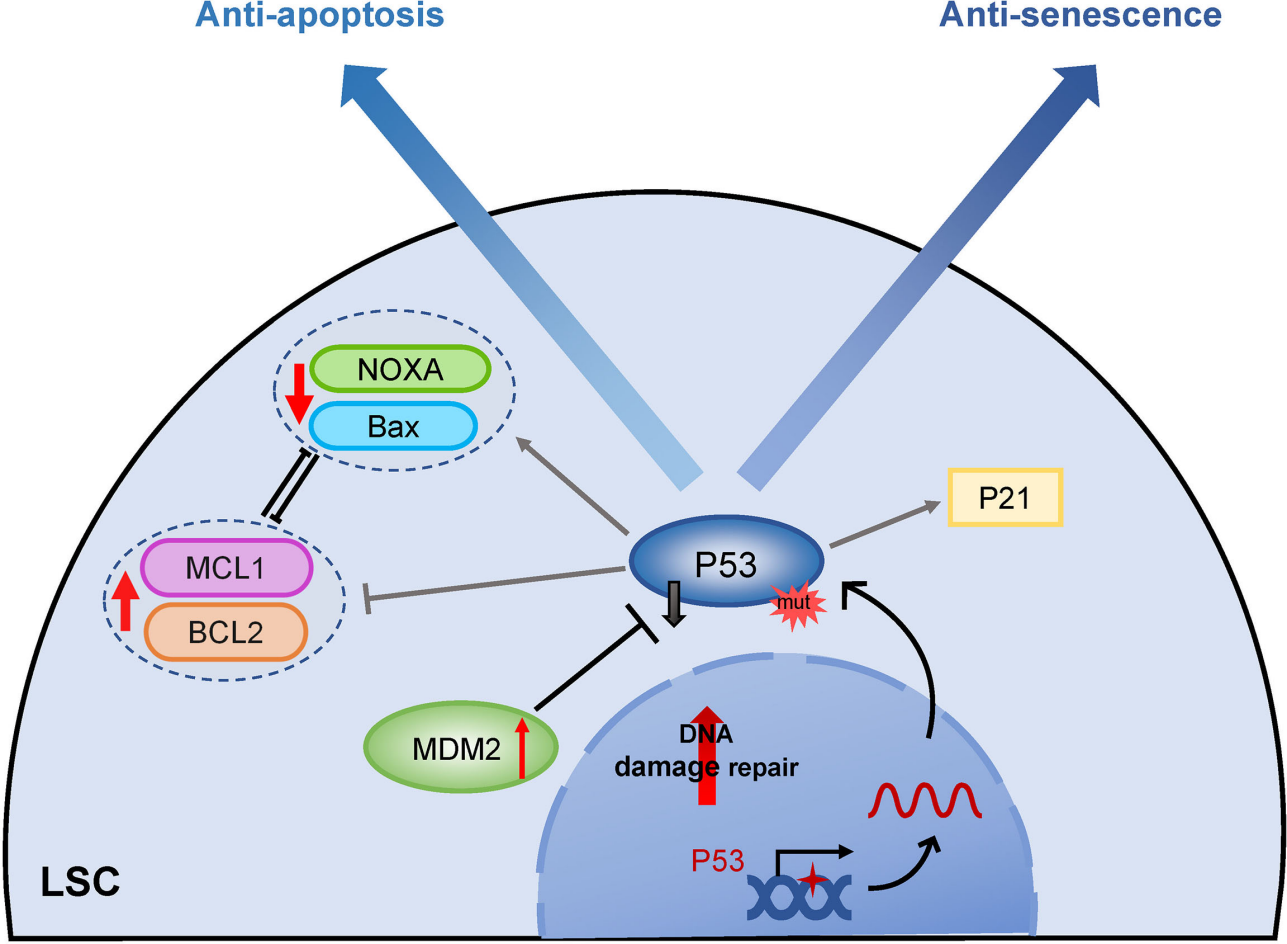
The role of TP53 in apoptosis and senescence. Mutations or inactivation of p53 plays an important role in both apoptosis and senescence resistance of LSCs. While LSCs with mutant P53 are unable to activate downstream P21 to enter the senescent process, they do not target apoptosis pathway proteins including induction of pro-apoptotic proteins like Bax, and apoptosis initiator groups like NOXA and repressing anti-apoptotic members BCL2 and MCL1, eventually fail to initiate apoptosis. MDM2, minute 2 homolog; BCL2, B-cell lymphoma 2; MCL1, myeloid cell leukemia sequence 1; Bax, Bcl-2-associated X protein; NOXA, proapoptotic BH3-only protein.

Mutation of the anti-oncogene p53 also has a great contribution to apoptosis resistance, and p53 plays a central role in the initiation of apoptosis in different physiological conditions (**Figure 4**) (109). Although the frequency of p53 mutation is low in newly diagnosed AML, the mutation rate is markedly elevated in therapy-related AML patients (110). Absent apoptosis signals by p53 gene mutation result in chemotherapy resistance (111). Furthermore, p53 may selectively activate the MDR-1 promoter, causing leukemia cells to develop multidrug resistance (112). AML patients with mutated p53 exhibit a poor response to chemotherapy and a poor prognosis. The finding suggested that restoring p53 activity through histone deacetylation could enhance the targeted chemotherapeutics in LSCs (113). PRIMA-1, a small molecule, has been reported to restore mutant p53 activity *via* binding to DNA and induction of apoptosis in p53-deleted AML, which enhances the sensitivity of chemotherapy drugs (50). In wild-type TP53 AML, overexpression of MDM2 (minute 2 homolog, a negative regulator of p53) leads to inactivation of p53 expression, which also exhibits apoptotic resistance. Indeed, several inhibitors targeting the MDM2-p53 axis to restore p53 function have shown broad anti-leukemia activities (48, 114). P53 activation promotes MCL1 degradation through downregulating the RAS/RAF/MEK/ERK signaling pathway, therefore, MDM2 inhibitor in combination with BCL2 inhibitor overcomes MCL1-mediated apoptotic resistance and shows synergistic lethality of relapsed/refractory AML in preclinical and clinical trials (115) (**Table 1**).

## Senescence Resistance of LSCs Involves Drug Resistance

The observations indicated that stress-induced activation of p38 mitogen-activated protein kinase (p38 MAPK), ROS, and DNA damage response (DDR) could induce HSCs senescence (116). However, a previous study found that LSCs had higher p38 MAPK activity, ROS levels, and increased accumulation of DNA damage but lower cellular senescence compared with HSCs (117). Wajapeyee et al. demonstrated that oncogenes induced senescence of HSCs rather than of LSCs (118). These investigations collectively suggested that senescent resistance mechanisms existed in LSCs. Ablain et al. were the first to identify the mechanisms involving arsenic trioxide in the treatment of acute promyelocytic leukemia (APL). Arsenic trioxide induced senescence through the promyelocytic leukemia gene (PML)-p53 pathway rather than by inducing APL cells apoptosis, which indicated that the induction of senescence was critical for the eradication of LSCs (49). Our recent data firmly demonstrated that lower expression of miR-34c-5p, a central regulator of cell senescence, was probably a significant factor involved in the resistance of LSCs senescence. Up-regulated miR-34c-5p in LSCs induces cellular senescence and promotes the eradication of LSCs in mouse models, which further supported the hypothesis (119).

Most chemotherapy drugs exert their therapeutic effect by inducing senescence of AML cells. However, recent studies have indicated that LSCs present multiple mechanisms of senescence resistance compared with HSCs, and also result in chemotherapy resistance. Until recently, these mechanisms have been poorly understood, for the following potential reasons (1) LSCs present the abnormal expression of both known and unknown molecules related to the senescent signaling pathway; and (2) LSCs activate DNA repair mechanisms leading to chemotherapy-induced DDR that fail to induce cell senescence.

Molecular abnormalities in the p53/p21 or p16/pRb axes, two fundamental signal pathways regulating cell senescence (120), could result in resistance of LSCs senescence. Mutational inactivation of p53 is a significant factor influencing senescence resistance and poor therapeutic outcomes in AML (**Figure 4**). The mutation rate of p53 in AML patients is less than 10%, and p53 mutation-positive AML patients are mostly treatment-related or are associated with cases of AML with a complex karyotype (110, 121, 122). P53 has been strongly associated with poor chemotherapy efficacy and prognosis of AML, whereas refractory and relapsed (R/R) LSCs are largely dependent on p53 mutation inactivation (112, 123). Currently, few reports have evaluated the expression of senescence regulatory molecules such as p16 and p21 in LSCs, which implicits a research direction to be explored in the future.

DNA damage is considered an important factor inducing cell senescence. Chemotherapy drugs induce DNA damage, and simultaneously DDR occurs in HSCs and LSCs (124). Interestingly, HSCs are more sensitive to chemotherapeutic agents than LSCs. Normal HSCs possess mechanisms able to inhibit DNA damage repair. DNA damage prolongs the S phase of the cell cycle and promotes DNA damage repair, but severe DNA damage can lead to cellular senescence (125). Hence, HSCs may progress to premature senescence induced by DNA damage, which can reduce the accumulation of harmful mutations in HSCs but also disrupts normal hematopoiesis. Conversely, LSCs are able to escape this mechanism, which does not appear to be associated with the phenomenon of premature senescence induced by chemotherapeutic drug-induced DNA damage, because the LSCs population lacks the signals inhibiting DNA damage repair (126). Thus, several DNA damage repair mechanisms are activated in LSCs treated with chemotherapy agents, which protect LSCs from chemotherapy-induced DNA damage and cell senescence (127, 128). Furthermore, sequential chemotherapy cycles also lead to depletion of the normal HSCs pool, and exacerbate the growth advantages of LSCs, resulting in R/R leukemia. Therefore, the restoration of senescence-initiated transmitting signals after chemotherapy-induced DNA damage may represent a novel approach for effectively eradicating AML. Evidence showing that the combined treatment of miR-34c-5p and chemotherapy facilitated LSCs senescence and elimination of LSCs *in vivo* preliminarily confirmed this hypothesis (119).

A more recent study found that following cytarabine chemotherapy, the enriched-senescent AML cells produced a senescence-associated secretory phenotype (SASP) by increasing NF-κB transcription through the ataxia telangiectasia and Rad3-related (ATR) activities, and regained stemness properties sufficient to restore leukemia (129). These effects may be mediated by changes in the SASP in a specific context. Further, it is important to understand how to eradicate senescent AML cells and overcome the adverse effects of SASP to prevent a recurrence.

## Metabolic Reprogramming-Induced Chemoresistance in LSCs

**Figure 5:**
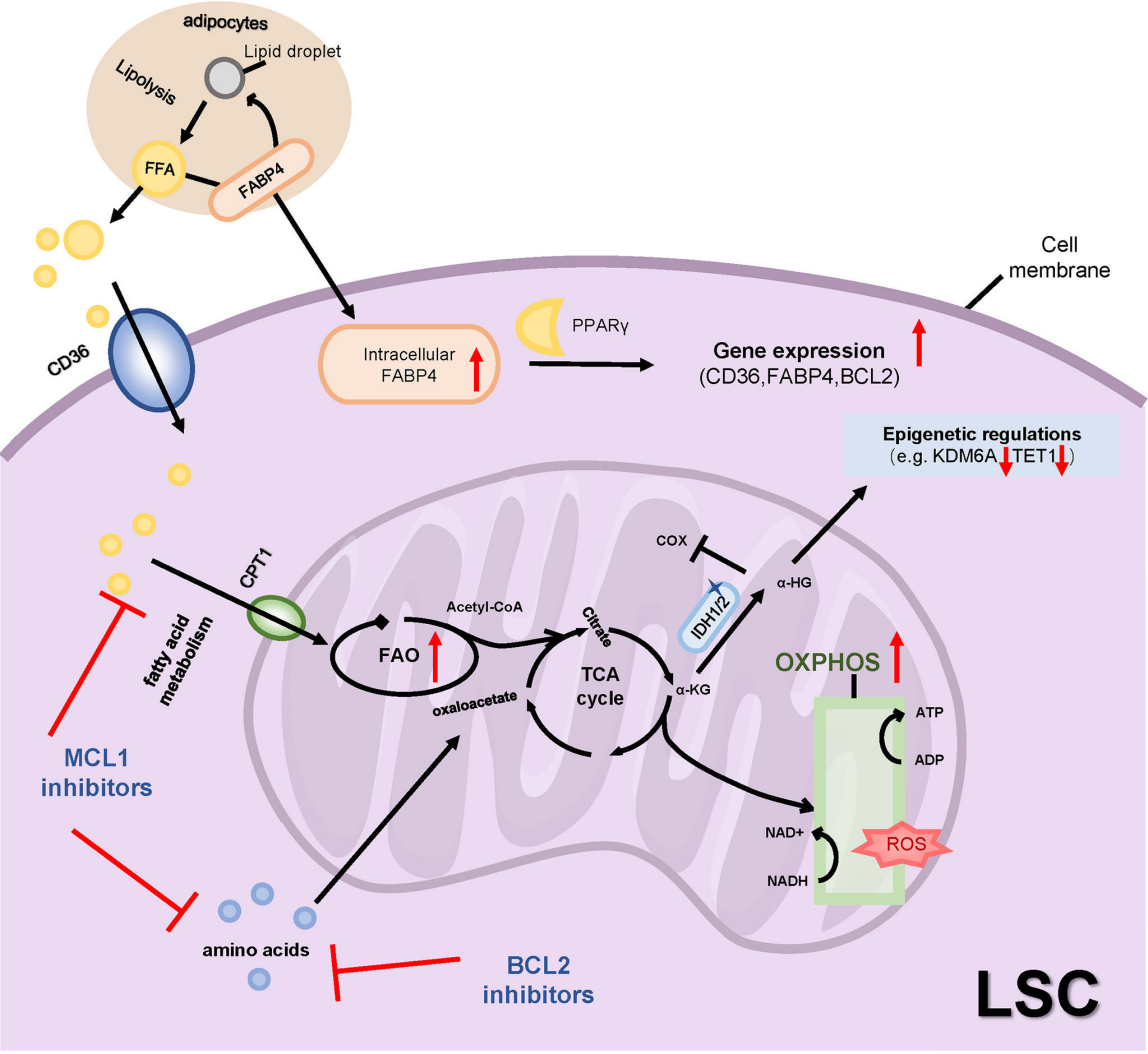
Metabolic alteration and drug resistance. LSCs are particularly dependent on OXPHOS, which utilizes amino acids and fatty acids rather than glucose as energy substrates to generate ATP. Therapy-resistant LSCs exhibit increased FAO through increasing fatty acid transporters such as CD36, CPT1 and FABP4. Inhibitions of MCL1 or BCL2 inhibit amino acid and fatty acid metabolisms to reduce OXPHOS. The mutant IDH1/2 catalyzes the conversion of α-KG to 2-HG, the latter regulates both epigenetic and metabolic changes. OXPHOS, oxidative phosphorylation; FAO, fatty acid oxidation; TCA, tricarboxylic acid cycle; FFA, free fatty acids; FABP4, fatty acid-binding protein-4; PPARγ, peroxisome proliferator-activated receptor γ; CPT1, carnitine O-palmitoyltransferase 1; α-KG, α-ketoglutaric acid; α-HG, 2-hydroxyglutaric acid; IDH1/2, isocitrate dehydrogenase 1 or 2; cox, cytochrome c oxidase; KDM6A, Lysine Demethylase 6A.

Enhanced glycolysis and an active truncated tricarboxylic acid (TCA) cycle have been described in many malignancies (130). The significant increase in AML blast cells leads to higher glucose uptake and the accumulation of a large amount of lactate, even under unlimited oxygen conditions, noted as the Warburg effect (131). The serum changes of 6 metabolites involved in glucose metabolism, including increased glycerol-3-phosphate, lactate, citrate, and decreased pyruvate, 2-oxoglutarate, 2-hydroxyglutaric acid (2-HG), have independent prognostic values in AML patients, in which poor prognosis was linked to accelerated glycolysis and activation of the TCA cycle (132). Additionally, HSCs reside in the hypoxic BM niche with tightly limited oxidative stress. HSCs specifically utilize glycolysis for energy generation while suppressing the influx of glycolytic metabolites into the mitochondria *via* pyruvate dehydrogenase kinases (PDKs) activity, which are downstream effectors of HIF-1α (133). Nevertheless, LSCs are particularly dependent on oxidative phosphorylation (OXPHOS) (63). OXPHOS utilizes amino acids, fatty acids, and glucose as energy substrates to generate ATP. It is more efficient to maintain LSCs survival in a hypoxic and nutrient-deprived microenvironment (**Figure 5**). Increased OXPHOS activity in LSCs has been associated with chemotherapy resistance. In primary human AML, despite the increased mitochondrial mass, LSCs display reduced spare respiratory capacity for OXPHOS compared to mature AML blasts and normal HSCs (134). It appears to be paradoxical that LSCs are maintained in a low ROS state because OXPHOS is prone to produce more ROS. Therefore, the metabolic stringency of LSCs suggests that they are susceptible to OXPHOS disturbances.

BCL2 is highly expressed in LSCs defined by low ROS levels. Venetoclax targets LSCs by inhibiting OXPHOS and impairs energy homeostasis. Jones et al. suggested that LSCs were uniquely dependent on amino acids that catabolize into the intermediates of the TCA to fuel OXPHOS in *de novo* AML patients. In relapsed AML patients, fatty acid catabolism was alternatively upregulated to maintain OSPHOS levels upon venetoclax plus azacitidine (ven/aza) treatment (135). Another study revealed that R/R AML patients achieved a worse response to ven/aza treatment because of elevated nicotinamide metabolism in LSCs which activated both amino acid and fatty acid metabolism in the TCA cycle to drive OXPHOS. Moreover, inhibition of nicotinamide phosphoribosyltransferase (NAMPT), the rate-limiting enzyme for the biosynthesis of nicotinamide adenine dinucleotide (NAD+), specifically targeted R/R LSCs by decreasing OXPHOS. Further, NAMPT inhibitors decreased glycolysis, but not OXPHOS, in AML blasts with high ROS levels, suggesting a metabolic heterogeneity of AML cells (136). In addition to the metabolic plasticity of LSCs, loss of BCL2 expression is another mechanism of venetoclax drug resistance. In particular, patients with monocytic AML are preferentially dependent on the anti-apoptotic protein MCL1 for OXPHOS activity (106). Inhibition of MCL1 showed a dual restriction on amino acid levels and fatty acid oxidation to reduce OXPHOS. Pre-clinical trials investigating multiple MCL1 inhibitors MIK665 (44), PRT1419 (45), AMG176 (46) and AMG397 (137) have been reported to exert anti-leukemia activity and have reported synergistic effects with venetoclax in murine models of AML (**Table 1**).

As described previously, ven/aza-resistant LSCs exhibit increased fatty acid oxidation (FAO) compared to ven/aza-sensitive LSCs, including L-carnitine and acyl-carnitines rather than the levels of fatty acids (138). BM adipocytes provide sufficient fatty acids for LSCs survival and proliferation. Inhibition of fatty acid transporters, such as fatty acid transporter CD36, fatty acid mitochondrial transporter CPT1, and fatty acid-binding protein 4 (FABP4), are other mechanisms that reduce FAO to overcome drug resistance. The expression of CD36 was increased in AraC-resistant LSCs and enhanced proliferative activity (63). Studies have shown that CD36^+^ LSCs were protected from chemotherapy damage by the microenvironment of gonadal adipose tissue (GAT). Drug-resistant LSCs secretes TNF-α, IL-1α, IL-1β, and CSF2 to promote GAT lipolysis and the release of free fatty acids (FFA) in the MLL-AF9 mouse model. CD36^+^ LSCs increased intracellular FAO levels by uptake of lipolytic free fatty acids (139). And deletion of CD36 in combination with ven/aza showed the potential to eliminate resistant LSCs. The CPT1 inhibitor, etomoxir, similarly decreased LSCs activity only in the presence of ven/aza, indicating that inhibition of both amino acid and fatty acid metabolism is necessary to target LSCs resistant to ven/aza treatment (138). BM adipocytes have higher FABP4 expression and promote lipid transport to support the activities of AML cells, and FABP4 can activate the downstream peroxisome proliferator-activated receptor (PPARγ) to further induce gene transcription of CD36, FABP4, and BCL2 in AML cells (140). These studies explain how adipose tissue and fatty acid transport support AML metabolism and are associated with drug resistance (**Figure 5**). In addition, the sphingolipid pathway is involved in the regulation of leukemia cell apoptosis, thus targeting sphingolipid metabolism also has considerable clinical significance (141). Xie SZ et al. demonstrated that S1PR3, a receptor for sphingosine-1-phosphate, was specifically overexpressed in the myeloid-like subset of LSCs. S1PR3 regulates myeloid differentiation, activates inflammatory and metabolic-related pathways through TNFα–NF-κB axis which disrupts LSCs stemness (142).

Mutations in isocitrate dehydrogenase 1 or 2 (IDH1/2) occur in 10 to 20% of the patients with AML and play an important role in the occurrence of AML (143). The mutant IDH1/2 catalyzes the conversion of α-ketoglutaric acid (α-KG) to 2-HG. The accumulation of the oncometabolite 2-HG regulates both epigenetic and metabolic changes in AML (**Figure 5**). 2-HG consistently inhibited the activity of cytochrome c oxidase (COX) and promoted the breakdown of L-glutamine, inducing the dependence of LSCs on BCL2, thus demonstrating the therapeutic sensitivity of venetoclax (51). Importantly, the US Food and Drug Administration (FDA) has approved inhibitors targeting mutant IDH1 or IDH2 to treat AML with relapses or resistance to other drugs. Clinical trials of ivosidenib and enasidenib, respectively mIDH1 and mIDH2 inhibitors, in newly diagnosed and R/R AML patients with IDH1/IDH2 mutations have improved chemotherapy response, and mutation-cleared AML patients had a longer duration of remission and a longer overall survival than patients without mutation clearance (3, 144–146). Other clinical trials reported that IDH1/2 inhibitors plus azacytidine achieved a higher complete response rate and mutation clearance and prolonged survival (147)(**Table 1**). But in patients with AML with simultaneous FLT3-ITD or RAS mutations, IDH1/2 inhibitors were less effective (148, 149). Mutations in receptor tyrosine kinase (RTK) pathway genes and IDH-related mutations were associated with resistance/relapse of ivosidenib (149). These prompt us to pay attention to the resistance mechanisms of IDH inhibitors and their combined application with other targeted inhibitors.

## Epigenetic Alternations Driving Drug Resistance of LSCs

**Figure 6:**
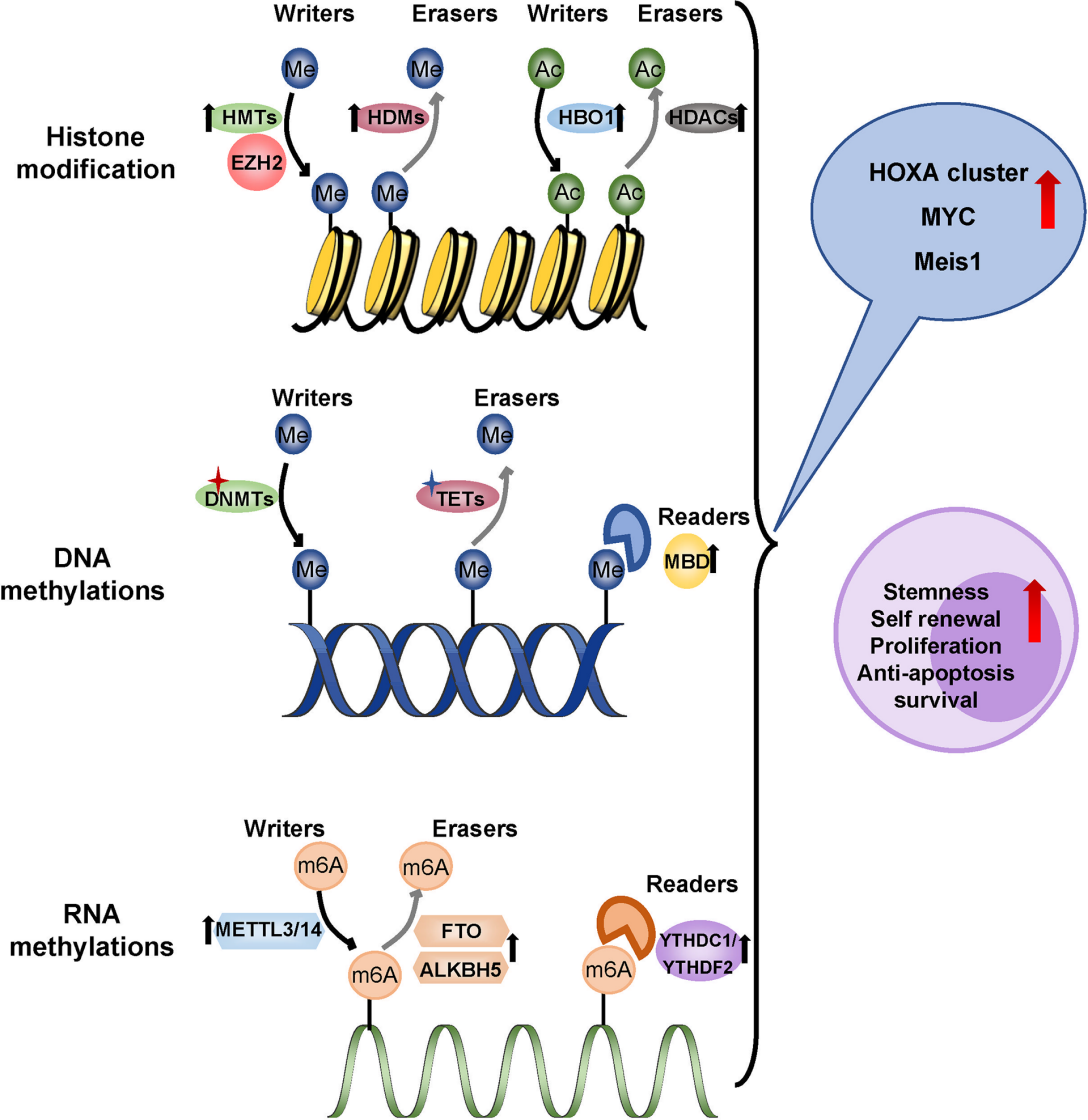
Epigenetic regulation in LSCs. Methylation operates at the DNA, RNA, and histone levels as a ‘reader’, ‘writer’ and ‘eraser’. LSCs showed a hypomethylated state which overexpressed the tumor-promoting gene like HOXA cluster, MYC and MEIS1. Overexpressed HBO1 in LSCs adds acetyl to histone H3K14 up-regulating HOXA gene transcription. Me, methyl; Ac, Acetyl; HMTs, histone methyltransferases; HDMs, histone demethylases; EZH2, zeste homolog 2; HBO1, lysine acetyltransferase 7; HDACs, histone deacetylases; DNMTs, DNA methyltransferases; TETs, ten-eleven translocation; MBD, methyl-CpG binding domain protein 2; m6A, N6-methyladenosine; METTL3/14, methyltransferase-like protein 3/14; FTO, fat mass and obesity-associated protein; ALBH5, ALKB Homolog 5YTHDC1/YTHDF2, YTH family proteins.

Driver mutations in leukemia often involve epigenetic genes, and subsequent epigenetic abnormalities promote the progression of AML. Specific epigenetic modifications of LSCs are linked to chemotherapy resistance and relapse. Methylation, the most common type of epigenetic modification in tumors, operates at the DNA, RNA, and histone levels as a “reader”, “writer” and “eraser” (**Figure 6**).

Aberrant DNA methylations are common molecular changes, including DNA methylation mutations and hypermethylation of CpG islands, which are associated with the prognosis of patients with AML (150). DNA hypermethylation generally inhibits gene expression, while hypomethylation leads to chromosome instability and abnormal gene activation (151). It was generally believed that the mutation of epigenetic factors was mainly involved in the conversion of HSPCs to pre-LSCs, and involved mainly DNA methyltransferase 3A (DNMT3A) and Tet methyl cytosine dioxygenase 2 (TET2), which made HSCs more prone to leukemogenesis (152). Pre-LSCs mutations that persist after chemotherapy were more likely to relapse of AML. DNMT3A mutations are frequently accompanied by FLT3 and NPM1 mutations in AML. The frequency of LSCs was increased in samples from patients with triple mutations in AML, indicating a poor prognosis (153). The deletion mutations of TET2 coexisted with mutations such as TP53, FLT3, and KRAS, cooperating to drive the occurrence of leukemia, which is related to a poor prognosis (154). Another DNA methylation reader, the methyl-CpG binding domain protein 2 (MBD2), maintained the quiescent state of LSCs by inhibiting the expression of cyclin-dependent kinase inhibitor 2C (CDKN2C) by hypermethylation (155). As a whole, LSCs showed a hypomethylated state rather than mutation dependence, which overexpressed the HOXA cluster, suggesting the possibility of resistance to hypomethylating agents (156).

In recent years, increasing studies have revealed the role of RNA epigenetic modification in the initiation and progression of AML. N6-methyladenosine (m6A) is the most frequent RNA epigenetic modification including METTL3/14 methylase as “writer” proteins, FTO/ALKBH5 demethylase as “eraser” proteins and YTH family proteins as “reader” proteins. Patients with higher m6A scores had a higher survival rate, while those with lower m6A scores had a poor prognosis. Methyltransferase 3(METTL3) increased the translation of downstream leukemia-related genes, such as MYC, BCL2, and PTEN mRNA, in an m6A-dependent manner, and depletion of METTL3 blocked proliferation, induced differentiation, and apoptosis in immunodeficient recipient mice (157). In addition, METTL3 recruitment by the transcription factor CEBPZ to the promoter of SP1, increased its mRNA methylation level, and promoted its protein translation by alleviating ribosomal stasis, which was essential for the maintenance of LSCs (158). STM2457, a METTL3 inhibitor, strongly reduced the frequency of LSCs, inhibited the implantation and progression of AML derived from patient xenografts, and prolonged the life span of recipient mice (159). METTL14 has also been found to play an important role in LSCs. METTL14 can enhance the stability of the oncogenic transcription factor MYB and MYC mRNA and promote translation to progress AML (160). The fat mass and obesity-associated protein (FTO) was the first m6A demethylase identified that removes m6A methylation modifications. FTO was more abundant in CD34^+^ AML cells. Knockdown of FTO in LSCs achieved a lower frequency of LSCs, impaired their self-renewal ability, and promoted apoptosis and differentiation by decreasing the expression of MYC and CEBPA in an m6A-dependent manner (161). Meanwhile, inhibition of FTO significantly reprogrammed the immune response by inhibiting the expression of the immune checkpoint gene LILRB4, making leukemia cells sensitive to T cell toxicity and overcoming hypomethylation agent-induced immune evasion (162). Additionally, ALKB Homolog 5 (ALKBH5) was significantly higher in LSCs derived from AML patients, which was related to a poorer prognosis. The deletion of ALKBH5 can significantly inhibit proliferation and impair the leukemia initiation potential in the immunodeficient mouse model (163). ALKBH5 acts through the KDM4C-ALKBH5-AXL signal axis in AML. KDM4C increased chromatin accessibility by recruiting MYB and Pol II to the ALKBH5 promoter and reducing H3K9me3. ALKBH5 subsequently promoted the expression of receptor tyrosine kinase (RTK) AXL, further activated downstream PI3K, MAPK, JAK/STAT, and NF-κB pathways, and played an essential role in the development and maintenance of LSCs (164). Furthermore, recent studies have shown that the m6A recognition protein YTHDF2 (YTH N6-Methyladenosine RNA Binding Protein 2) promoted the stemness maintenance of AML LSCs. The deletion of YTHDF2 improved apoptosis to selectively eliminate LSCs (165). Recently, it has been discovered that YTH Domain Containing 1 (YTHDC1) was the top essential m6A reader in AML from a genome-wide CRISPR screen. YTHDC1 enhanced MYC expression and reduced degradation to maintain leukemogenesis. YTHDC1 knockdown also reduced LSCs proliferation, increased differentiation, and delayed the development of leukemia (166).

Histone modification is an essential form of epigenetic regulation. Histone methyltransferases (HMTs) and histone demethylases (HDMs) regulate methylation at different histone locations. The Histone-H3 lysine-79 (H3K79) methyltransferase DOT1L and H3K4 demethylases KDM1A play a key role in the occurrence and development of LSCs harboring the MLL rearrangement by up-regulating stem cell-related genes HOXA and Meis (167, 168). In the primary AML cells, the KDM1A inhibitors inhibit PI3K pathway and activate the MEK pathway, further inhibition of KDM1A sequential the MEK inhibitor significantly promotes leukemia cell apoptosis especially in M5 leukemia, which was associated with protein phosphorylation and RAS mutation (169). The DOT1L inhibitor EPZ5676 (170) and KDM1A inhibitor ORY-1001 (52), respectively, inhibited the proliferation and differentiation of MLL-LSCs and increased the survival of transplanted mice. Nuclear receptor binding SET Domain Protein 1 (NSD1) encodes mono- and di-methylation of H3K36. The NUP98-NSD1 fusion activated HOX gene through H3K36 hypermethylation for leukemogenesis, and was identified as a high-risk factor for AML (171). A recent study reported that targeting inhibitor of NSD1, BT5, reduced the expression levels of HOX cluster and MEIS1 and impaired the colony formation of primary AML cells (172). And inhibition of NUP98-NSD1 reduced leukemia burden and prolonged survival in NUP98-NSD1 patient-derived xenograft model (172, 173).

Other histone methylation modifications at methyl residues include H3K27 methyltransferase component zeste homolog 2 (EZH2) and H3K27 demethylase KDM6B. EZH2 is a member of the Polycomb inhibitory complex 2 (PRC2), which acts as a transcriptional inhibitor (174). Low levels or loss of the EZH2 protein are associated with chemoresistance and a poor prognosis (175). However, with the advanced evolution of AML, EZH2 may play the opposite oncogenic function, which demonstrates the dependence on tumor stage and context (176). Thus, targeted EZH2 therapy should consider leukemia status. 3-Deazaneplanocin A (DZNep), an inhibitor of EZH2 and H3K27me3, targeted LSCs to induce apoptosis (177). Overexpression of KDM6B is correlated with poor OS in AML. GSK-J4, a KDM inhibitor, decreased the level of the cAMP response element-binding protein (CREB) and inhibited the proliferation of LSCs (178). However, KDM6B also maintained self-renewal and quiescence of the HSCs (179). This may limit the clinical translation of GSK-J4.

Histone modification also includes histone acetylation. Generally, increased acetylation is related to up-regulating transcription activity, while decreased acetylation is related to inhibition of gene expression (**Figure 6**) (151). The MYST acetyltransferase HBO1 and acetylation of histone H3K14, upregulate the HOXA family of genes in MLL-LSCs, which are essential for the maintenance of LSCs. WM-3835 exhibits a remarkable anti-leukemic effect *in vitro* by the reduction of H3K14ac levels (180). The abnormal recruitment of histone deacetylase (HDAC) proteins into cancer-causing fusion proteins such as AML1-ETO, CBFB-MYTH11, PML-Rα and MLL plays an important role in the development of leukemia (181). However, clinical results have shown that HDAC inhibitors are not effective as a monotherapy for AML, and *in vitro* experiments have shown that HDAC inhibitors and DNMT inhibitors cooperate to induce the re-expression of silent genes in LSCs (53). Phase I clinical trial showed that panobinostat, a HDAC inhibitor, combined with intensive chemotherapy induced CR/incomplete count recovery (Cri) in 8 elderly AML patients (54). The reversibility of epigenetic modification provides an opportunity to personalize AML with specific epigenetic inhibitors.

## The Protection Mechanisms of LSCs Within the BM Microenvironment

**Figure 7:**
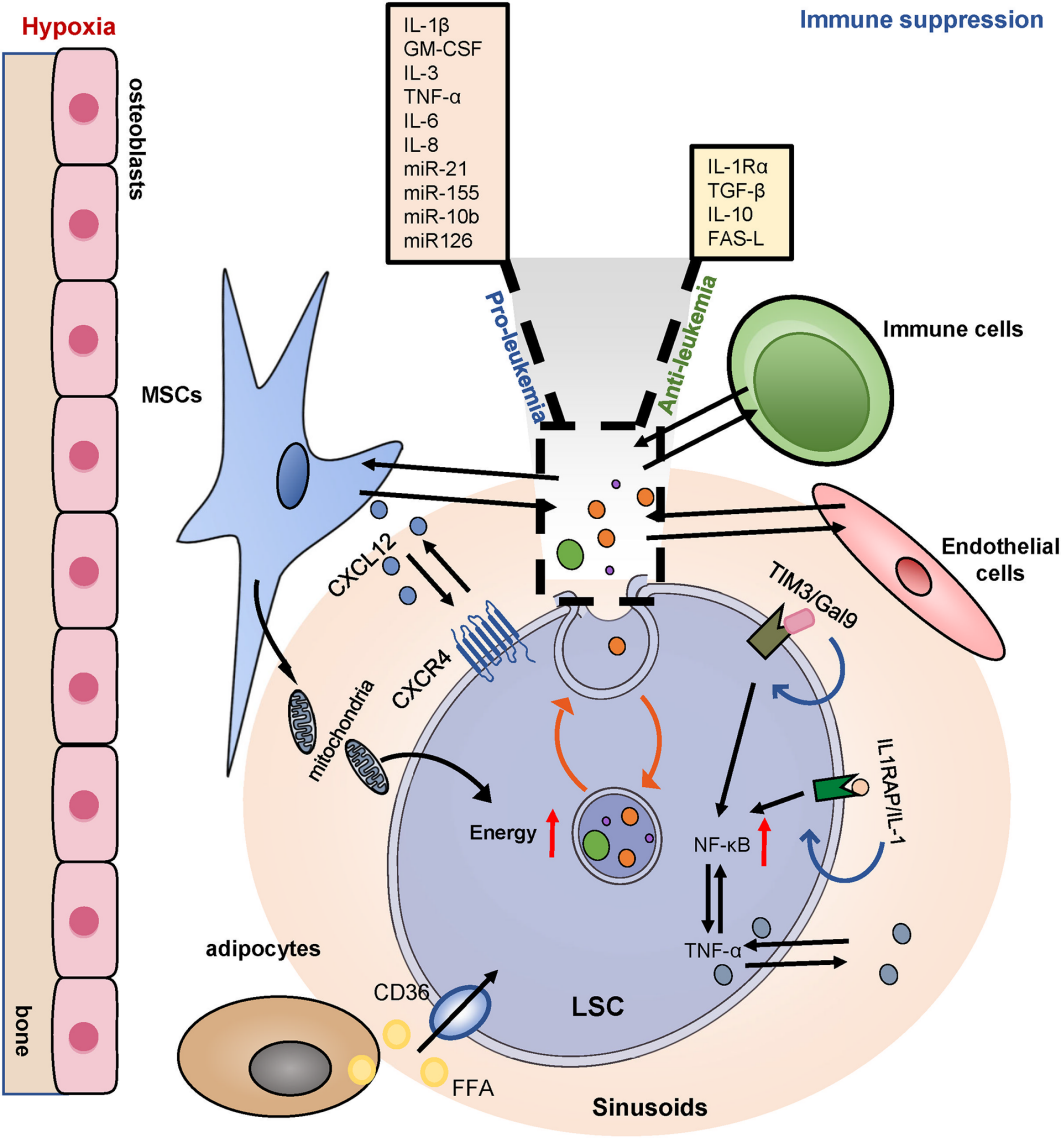
BM niches and drug resistance. LSCs utilize the BM niches for increased survival. LSCs highly express CXCR4, binding to its ligand CXCL12, positioned in a hypoxic area. BMSCs also transfer mitochondria to provide additional energy for the survival of LSCs. Adipocytes provide sufficient fatty acids for LSCs metabolic reprogramming. LSCs globally induce immune tolerance *via* upregulating TIM3 and inducing multiple chemokines and an inflammatory secretome and thereby promoting leukemia progression. BM, bone marrow; MSCs, mesenchymal stromal cells; CXCR4, CXC chemokine receptor-type 4; CXCL12, CXC motif chemokine ligand 12; IL1RAP, interleukin-1 co-receptor; TIM3/Gal9, T cell immunoglobulin and mucin protein 3/galectin-9; NF-κB, nuclear factor κB. EVs, extracellular vesicles.

Chemotherapy-resistant LSCs home and remodel the BM niche to allow leukemia survival, thus inhibiting normal hematopoiesis. The complex crosstalk between LSCs and their microenvironment contributes to LSCs survival, treatment resistance, and recurrence (**Figure 7**). Targeting the LSCs microenvironment is a critical avenue for the treatment of AML.

BMSCs play a key role in remodeling the leukemia niche. The CXCR4/CXCL12 axis plays a central role in the regulation of LSCs-BMSCs interactions. Blocking the interaction between CXCR4/CXCL12 effectively compromised the homing of LSCs into the BM niche and rendered leukemia cells sensitive to chemotherapy (55). The BMSCs transferred mitochondria to provide additional energy for the survival of both the AML blast cells and the LSCs. And recipient LSCs were resistant to cytarabine-induced apoptosis and had a greater potential for leukemia initiation (182). Furthermore, the accumulation of Nestin^+^ BMSCs in the bone marrow of AML was critical for the viability and proliferation of LSCs *in vitro* and *in vivo*, Nestin^+^ BMSCs induced chemoresistance by increasing energy production and glutathione-peroxidase (GPX) activity in LSCs (183). BMSCs protect AML cells from chemotherapy by increasing stem-type signaling pathways, such as the Notch and Wnt pathways, and by inhibiting apoptosis (184). In addition, senescent BMSCs increased the survival and proliferation of AML in the form of cytokine secretion (185).

The formation and maintenance of the AML niche depend on the imbalance of the cytokine profile. LSCs existed in a pro-inflammatory environment that was conducive to the survival and proliferation of LSCs. Autocrine tumor necrosis factor α (TNF-α) of LSCs activated NF-κB activity to form the NF-κB/TNF-α positive feedback loop (186). Similarly, LSCs highly expressed the interleukin-1 co-receptor IL1RAP, while deletion of IL1RAP inhibited the stemness of LSCs and increased apoptosis (187). Researchers have found a specific overexpression of T cell immunoglobulin and mucin protein 3 (Tim-3) in LSCs. In addition, the formation of the Tim-3/Gal-9 autocrine loop activated NF-κB and β-catenin pathways to support the self-renewal and survival of AML cells (56). Treatment with anti-Tim-3 antibodies not only reduced LSCs, but also regulated immune balance to prevent AML from immune evasion (188). In BM, several cytokines and soluble factors have been shown to influence the survival and growth of leukemia cells. For example, the pro-inflammatory cytokines IL-1β, GM-CSF, IL-3, TNF-α, and IL-6 appear to promote the proliferation of AML cells, while anti-inflammatory molecules such as IL-1Rα, TGF-β and IL-10 exert inhibitory effects (189). The specific functions of cytokines depend on the crosstalk of multiple complex molecules within the microenvironment.

Extracellular vesicles(EVs), classified into exosomes, microvesicles, and apoptotic bodies, also regulate the interaction between LSCs and the BM niche (190). A growing number of studies have shown that EVs played a key role in drug resistance and regulating immune response regulation. EVs derived from AML bone marrow-derived MSCs promote AML cell proliferation, migration and protect AML cells from treatment with AraC (191). BMSCs secrete exosomes and IL-8 to promote the resistance to etoposide in the AML stem cell line KG1a (192). Chemoresistant AML cells were able to induce increased expression of MRP-1 in chemosensitive AML cells through EVs to enable them to be resistant to chemotherapy drugs (193). EVs contain higher levels of known clinical risk factors, such as TGF-β1, miR-21, miR-155, miR-10b (194, 195). Similarly, exosomes carry Fas ligand (FAS-L), NPM1, FLT3, matrix metallopeptidase 9 (MMP9), insulin-like growth factor type 1 receptor (IGF1-R), CXCR4, and chaperones to alter the BM microenvironment and to improve the survival of leukemia cells, especially LSCs (196, 197). Overall, EVs can directly or indirectly alter the signaling pathways, or increase the mitochondrial content, leading to the proliferation, survival, and drug resistance of AML. Therefore, targeted blocking of EVs in the crosstalk between LSCs and the supportive microenvironment may be considered a promising therapy.

## Conclusions

Multiple intracellular and extracellular mechanisms are the root of drug resistance and relapse in AML stem cells, and influence the dormancy, resistance to apoptosis and senescence, epigenetic modification, metabolic reprogramming, and protection of the BM microenvironment. Although corresponding targeted drugs have been developed and are being applied in the clinic, it remains difficult to eradicate LSCs. We are now convinced that AML might alter leukemic clones during treatment, which results in tumor progression. LSCs were located at the top of the clonal heterogeneity, and their clonal diversification is accelerated by the tumor mutation burden (26). AML clone evolution is divided into linear and branching patterns. The former stepwise acquires clonal hematopoiesis (CH)-type mutations and AML-related mutations. The latter parallelly acquires CH-type mutations and AML-related mutations in different cell populations (198). CH is prominent in AML clone evolution, and is assumed to represent a pre-malignant state. DNMT3A, TET2, and ASXL1 (“DTA”) mutations prevalently occur early in pre-leukemia hematopoietic stem and progenitor cells (HSPCs), persist for a long time and are not considered to be associated with poor prognosis. The acquisition and persistence of other CH-type mutations like TP53, IDH1/2 are related to an increased risk of AML relapse (199). AML transformation occurs with acquirement of AML-related mutations like FLT3, NPM1, NRAS/KRAS. The relapse of LSCs clones derives from persistent clonal evolution or new sub-clonal structures post-chemotherapy. Mutations in AML can persist after treatment induction, or new types of AML mutations may appear after chemotherapy, eventually leading to the recurrence of leukemia (20) (**Figure 1**). LSCs are the root of drug resistance and are prone to the co-existence of multiple mechanisms and crosstalk with the BM microenvironment.

In general, treatment agents must be adjusted to target altered LSCs clones. Future treatment strategies have been proposed to address the dynamics of LSCs clones; thus, real-time monitoring of minimal residual disease (MRD) and timely adjustment of treatment regimens are critical. Finding more LSCs-specific therapeutic targets that can avoid affecting normal hematopoiesis. Furthermore, chemotherapy can induce multiple drug-resistant LSCs clones, and consequently, it is critical to focus on the elimination of naïve LSCs that can minimize the formation of polyclonal LSCs. Regarding relapsed drug-resistant LSCs, combined therapy is necessary due to the different mechanisms of drug resistance. Ultimately. Single-cell combined with multi-omics sequencing will contribute to the specific identification and the discovery of key mechanisms of LSCs. Preliminary clinical trials have shown that the combination of novel agents or with traditional chemotherapeutic agents increased clinical activity, which was beneficial in overcoming primary resistance and second resistance in AML.

## Author Contributions

JN and DP reviewed the literature and wrote the manuscript, LL provided concept and guidance and approved the final version to be published. All authors contributed to the article and approved the submitted version.

## Funding

The study was supported by grants from the National Natural Science Foundation of China (Nos. 81973999, 81900188, 81770192, 81370660), and the Wuhan Science and Technology Program, China (No. 2017060201010180).

## Conflict of Interest

The authors declare that the research was conducted in the absence of any commercial or financial relationships that could be construed as a potential conflict of interest.

## Publisher’s Note

All claims expressed in this article are solely those of the authors and do not necessarily represent those of their affiliated organizations, or those of the publisher, the editors and the reviewers. Any product that may be evaluated in this article, or claim that may be made by its manufacturer, is not guaranteed or endorsed by the publisher.
